# Paraneoplastic dermatomyositis with atypical features associated with a solid pseudopapillary pancreatic neoplasm

**DOI:** 10.1007/s10067-026-07928-z

**Published:** 2026-02-01

**Authors:** N. A. Uribe-Ruíz, D. A. Domínguez-Guzmán, J. C. Jaramillo-Álvarez, A. F. Vargas-Camacho, C. Pineda, M. Restrepo-Escobar, C. H Muñoz-Vahos, L. A González-Naranjo, A. L. Vanegas-García

**Affiliations:** 1 Rheumatology Group, Department of Internal Medicine, Universidad de Antioquia, Calle 64 #51D - 154, Medellín, Colombia; 2 Hospital San Vicente Fundación, Medellín, Colombia; 3 Neurology Resident, Universidad CES, Medellín, Colombia; 4 Hospital Alma Máter de Antioquia, Universidad de Antioquia, Medellín, Colombia; 5 Hospital San Juan de Dios de Yarumal, Antioquia, Colombia; 6 Asociación Colombiana de Reumatología, Bogotá, Colombia

**Keywords:** Dermatomyositis, Generalized subcutaneous edema, Ovoid palatal patch, Pseudoangioedema, Solid pseudopapillary pancreatic tumor

## Abstract

Dermatomyositis (DM) is an autoimmune inflammatory myopathy that may occur as a paraneoplastic syndrome, most commonly associated with ovarian, lung, and gastrointestinal malignancies. Solid pseudopapillary pancreatic tumor is a rare neoplasm with low malignant potential, and to our knowledge only one case has previously been reported in association with DM. We present the case of a 21-year-old woman with six months of proximal muscle weakness, dysphagia, and cutaneous lesions characteristic of DM, including a heliotrope rash, shawl sign, and V-sign. She also exhibited atypical features, such as pseudoangioedema, an ovoid palatal patch, and generalized subcutaneous edema. Laboratory studies showed elevated muscle enzyme levels and positivity for anti–TIF1γ antibodies. Imaging revealed a solid–cystic pancreatic mass, which was histologically confirmed to be a solid pseudopapillary tumor. Following surgical resection, muscle enzyme levels normalized and cutaneous manifestations improved, although proximal quadriparesis persisted (Fig. 1). This case expands the spectrum of neoplasms associated with DM and highlights the relevance of atypical cutaneous manifestations and anti–TIF1γ antibodies as markers warranting surveillance for occult malignancy. The clinical improvement following tumor resection further supports a paraneoplastic association.

**Table:** 

**Key Points** • * Atypical cutaneous findings—such as pseudoangioedema, ovoid palatal patch, and generalized subcutaneous edema—may serve as clinical indicators of paraneoplastic dermatomyositis. * • * The coexistence of dermatomyositis with a solid pseudopapillary pancreatic tumor represents an exceedingly rare association. * • *This case study underscores the significance of incorporating rare pancreatic tumours in the list of malignancies for patients diagnosed with dermatomyositis and anti-TIF1γ antibodies.* • * Improvement of cutaneous lesions and normalization of muscle enzymes after tumor resection reinforce the causal link between the neoplasm and dermatomyositis. *

**Key Points**

• * Atypical cutaneous findings—such as pseudoangioedema, ovoid palatal patch, and generalized subcutaneous edema—may serve as clinical indicators of paraneoplastic dermatomyositis.
*

• * The coexistence of dermatomyositis with a solid pseudopapillary pancreatic tumor represents an exceedingly rare association.
*

• *This case study underscores the significance of incorporating rare pancreatic tumours in the list of malignancies for patients diagnosed with dermatomyositis and anti-TIF1γ antibodies.*

• * Improvement of cutaneous lesions and normalization of muscle enzymes after tumor resection reinforce the causal link between the neoplasm and dermatomyositis.
*

## Introduction

Idiopathic inflammatory myopathies (IIM) are a heterogeneous group of diseases with variable clinical manifestations. With advances in tools for characterizing immunologic, genomic, and transcriptomic profiles, classification into distinct subtypes has become possible [1]. DM is one such subtype and may present as a primary disease or as a paraneoplastic manifestation. It may occur concurrently with cancer, precede its diagnosis, or even arise after malignancy is identified [2, 3].

To determine malignancy risk in IIM, the International Myositis Assessment and Clinical Studies Group (IMACS) developed a consensus based on clinical characteristics and autoantibody profiles [2]. Although it guides risk-based screening strategies, it remains imperfect; even variables strongly associated with malignancy—such as anti–TIF1γ antibodies—may have variable associations [3]. Similarly, atypical dermatologic findings—including the ovoid palatal patch, pseudoangioedema, and generalized subcutaneous edema—should prompt a thorough malignancy evaluation when present, although they are not exclusive to cancer-associated disease [4–7].

The types of cancer most commonly associated with DM and anti–TIF1γ antibodies generally correspond to the most prevalent tumors according to age and sex [8], although they may also include exceptionally rare malignancies [9].

**Fig. 1:**
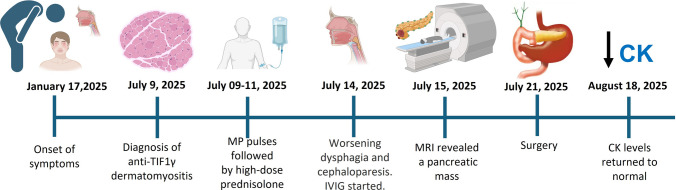
Clinical chronology. MP: methylprednisolone, IVIG: intravenous immunoglobulin, MRI: magnetic resonance imaging, CK: creatine kinase. Created with Biorender.com

This case-based review highlights infrequent cutaneous manifestations in the context of DM with anti–TIF1γ antibodies and their association with a very low-prevalence neoplasm Fig. 1.

## Case report

**Fig. 2:**
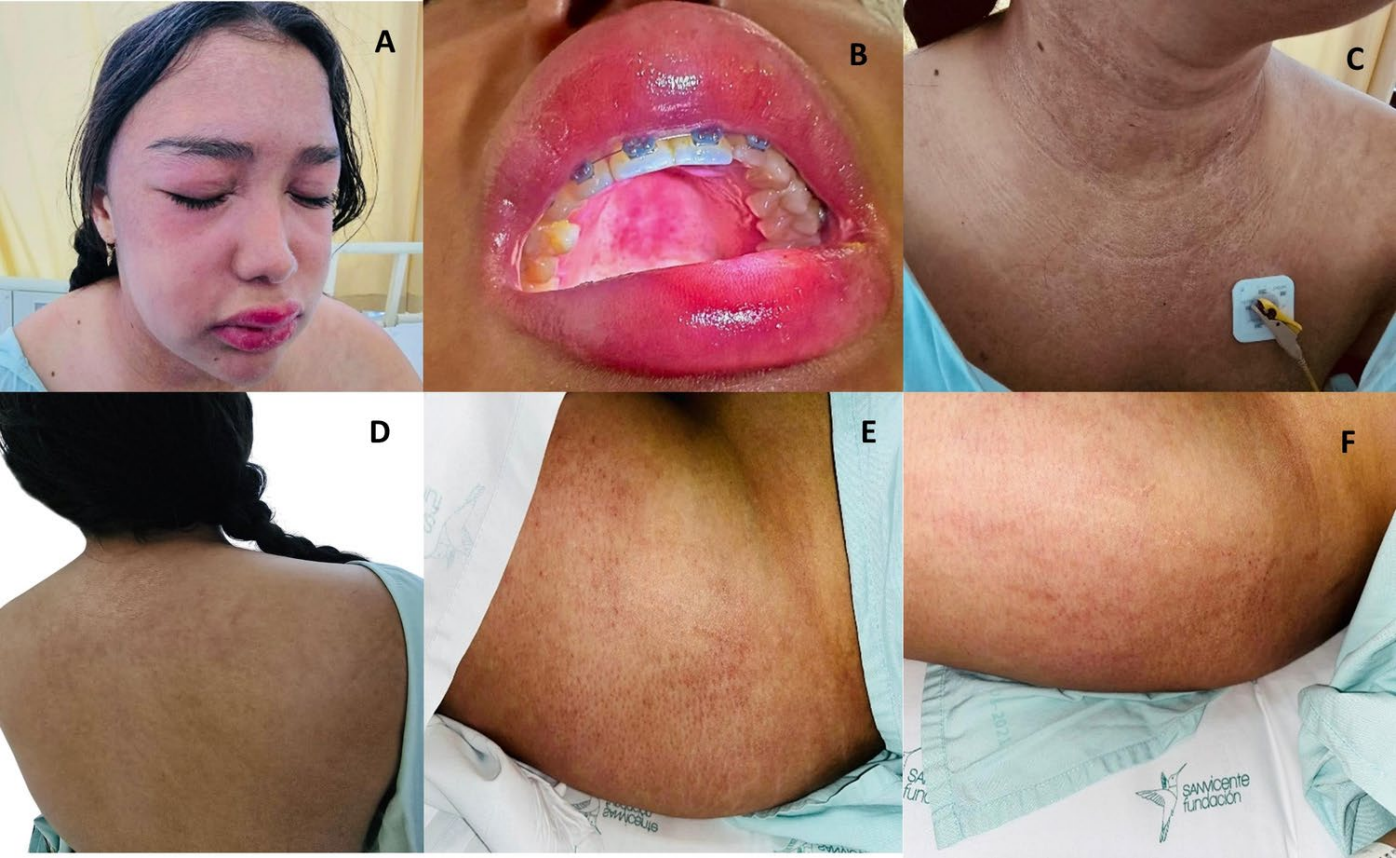
Dermatologic findings. **A**. Heliotrope rash and pseudoangioedema. **B**. Ovoid palatal patch and pseudoangioedema. **C**. Poikiloderma and V-sign. **D**. Poikiloderma and shawl sign. **E–F**. Bilateral holster sign

A 21-year-old woman with no significant medical history presented with a six-month history of progressive proximal muscle weakness of 3/5 on Medical Research Council (MRC) scale, dysphagia, and cutaneous lesions consistent with DM, including a heliotrope rash, shawl sign, V-sign, poikiloderma, and holster sign, along with pseudoangioedema and an ovoid palatal patch (Fig. 2).

Initial laboratory assessment showed marked elevation of muscle enzymes, including creatine kinase (CK) at 1,267 U/L (reference range [RR]: 34–145 U/L), lactate dehydrogenase (LDH) at 1,044 U/L (RR: 120–246 U/L), and aspartate aminotransferase (AST) at 157 U/L (RR: 14–35 U/L). Immunological testing revealed a high-titer antinuclear antibody (ANA) of 1:640 with a fine granular pattern (AC-4), while anti-dsDNA and extractable nuclear antigen (ENA) antibodies were negative. Complement levels remained within normal limits (C3: 106.8 mg/dL; C4: 18.6 mg/dL).

**Fig. 3:**
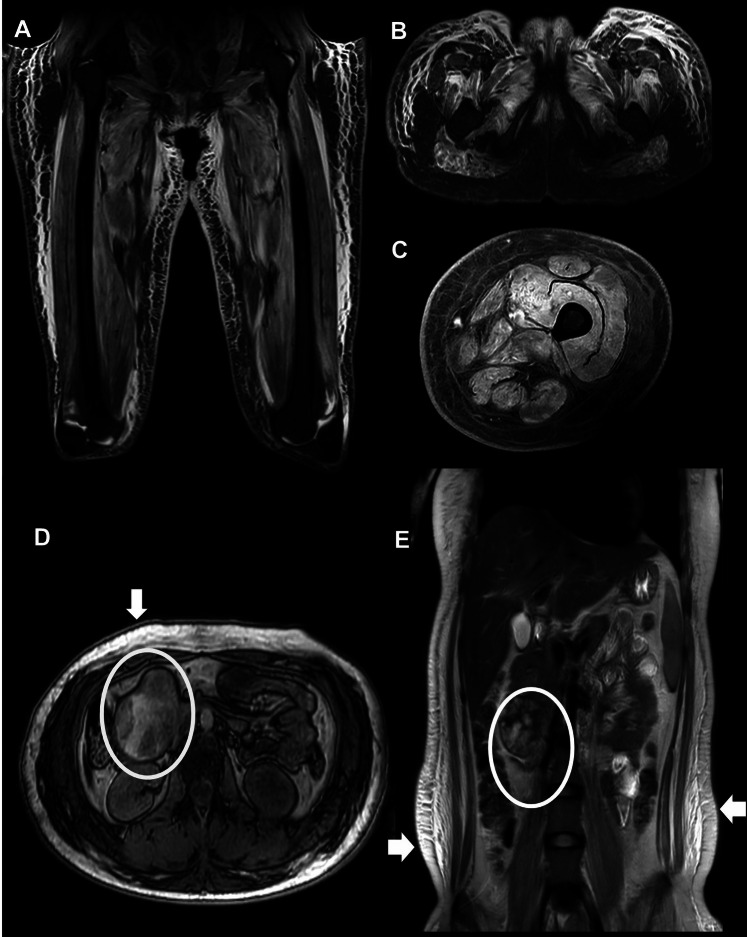
Non–contrast-enhanced MRI of the thighs. **A**. Coronal view. **B-C**. Axial view. Asymmetric and diffuse muscle edema involving the anterior, medial, and posterior compartments. Contrast-enhanced abdominal MRI. **D**. Axial view. **E**. Coronal view. Solid–cystic mass in the pancreatic head and uncinate process (white circles) and generalized subcutaneous edema (white arrows)

The extended myositis panel was strongly positive for anti-TIF1γ antibodies. Non-contrast magnetic resonance imaging (MRI) of the thighs revealed diffuse, asymmetric muscle edema across the anterior, medial, and posterior compartments. These findings were accompanied by subfascial and perifascial fluid collections, along with significant subcutaneous tissue edema (Fig. 3A-C). Given the concordance between clinical manifestations, serological markers, and imaging findings, a diagnosis of dermatomyositis was established; consequently, further invasive procedures, such as electromyography or muscle biopsy, were deemed unnecessary.

She was initially treated with methylprednisolone pulses (500 mg every 24 h for three days), followed by high-dose prednisolone (60 mg every 24 h). Subsequently, she developed muscle strength worsening (MRC grade 2/5) with severe dysphagia and cephaloparesis, prompting administration of intravenous immunoglobulin (IVIG) 60 g per day for two days.

**Fig. 4:**
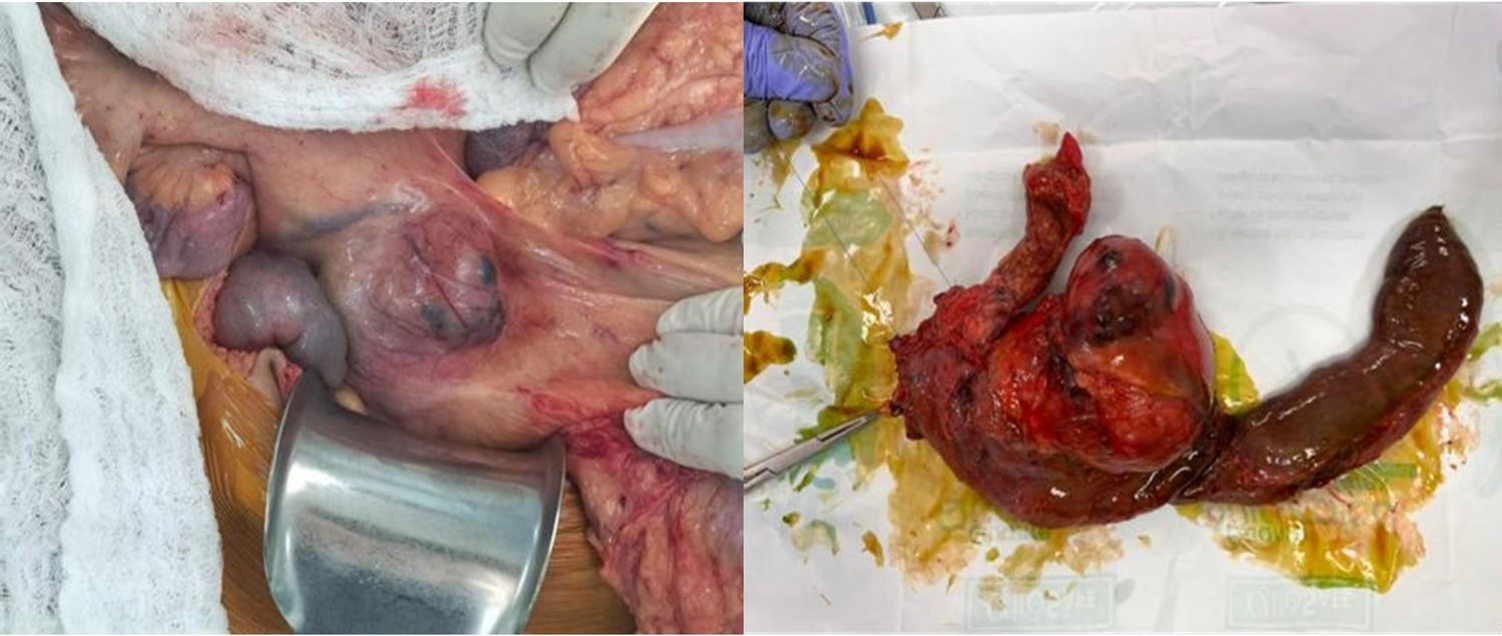
Surgical specimen from proximal pancreaticoduodenectomy. A lobulated pancreatic mass is observed adhering to the wall of the duodenum

**Fig. 5:**
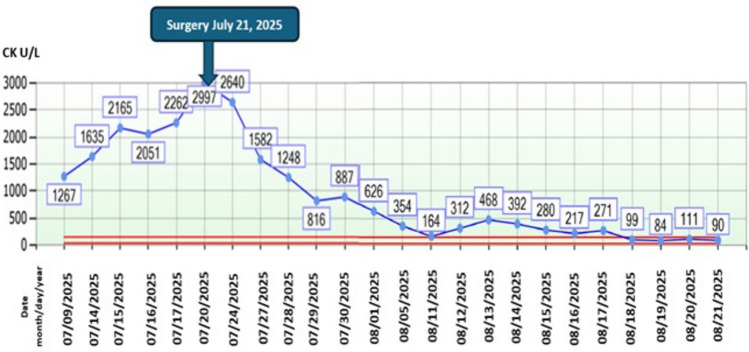
Creatine kinase (CK) levels from admission to tumor resection on July 21, 2025

The patient was classified as high-risk for idiopathic inflammatory myopathy (IIM)-associated malignancy, consequently, a comprehensive screening including both basic and enhanced cancer panels was conducted. All investigations were unremarkable, with the exception of a contrast-enhanced abdominal CT, which revealed generalized subcutaneous edema and a 4.9 × 6.9 × 5.2 cm solid–cystic lesion in the pancreatic head and uncinate process (Fig. 3D-E), highly suggestive of a solid pseudopapillary neoplasm. The patient underwent a proximal pancreaticoduodenectomy with complete R0 resection (Fig. 4); histopathological analysis confirmed the diagnosis. Postoperatively, clinical follow-up showed rapid normalization of muscle enzymes (Fig. 5) and dermatological recovery. However, while motor strength partially improved (MRC grade 3/5), refractory dysphagia persisted, requiring gastrostomy for nutritional support.

Her hospitalization was further complicated by aspiration pneumonia and ventilatory failure due to *Mycobacterium tuberculosis* infection, confirmed on bronchoalveolar lavage. Glucocorticoids were tapered, antituberculous therapy was initiated, and additional IVIG doses were administered every three weeks. After a four-month hospitalization, she was discharged with a rehabilitation plan and a schedule monthly IVIG regimen, however, two months post-discharge, the patient was admitted to the local hospital with no signs of life (dead on arrival).

## Discussion

DM is a heterogeneous disease with a wide spectrum of cutaneous manifestations, ranging from classic features—such as Gottron papules, heliotrope rash, and shawl/V-signs—to less common findings that may provide insights into phenotype and prognosis [6], including an ovoid palatal patch, pseudoangioedema, and generalized subcutaneous edema.

The ovoid palatal patch typically presents as a violaceous, symmetric, well-demarcated plaque crossing the midline of the hard palate; it is non-ulcerated and usually asymptomatic [10, 11]. In a cohort of 45 DM patients, Bernet et al. reported this finding in 40%, showing significant associations with amyopathic DM, anti–TIF1γ positivity, and increased malignancy risk, including more advanced-stage tumors and shorter intervals between DM onset and cancer diagnosis [4].

Pseudoangioedema is an uncommon but well-recognized manifestation of DM. It presents as non-allergic inflammatory edema that mimics true angioedema but without pruritus, urticaria, or airway involvement. It typically affects the face or periorbital region and may be accompanied by violaceous erythema or other classic DM lesions [5, 12]. The pathophysiology involves immune-mediated vasculopathy and increased capillary permeability due to endothelial injury and immune complex deposition [6]. Pseudoangioedema may appear at disease onset or during the course of DM and is sometimes associated with paraneoplastic forms linked to anti–TIF1γ antibodies, as observed in this patient. It does not respond to antihistamines or epinephrine but improves with immunosuppressive therapy [12, 13].

Generalized subcutaneous edema is also rare. Proposed mechanisms include increased capillary permeability due to immune complex deposition, vascular injury, inflammation in adjacent muscle, vasculitis, or lymphatic obstruction [13]. Overexpression of vascular endothelial growth factor (VEGF) in DM muscle suggests that VEGF-induced vascular permeability contributes to this phenomenon, although serum VEGF levels are usually normal [14]. These cases typically present with severe, treatment-resistant disease, predominantly affecting the extremities [14, 15]. These findings should be considered red flags warranting exhaustive malignancy screening [10, 12, 14–16]. Larger cohorts are needed to confirm their specificity.

Pancreatic cancer accounts for ~ 1.54% of DM-associated malignancies [17–20], and among the few reports with histology, most cases are adenocarcinomas [18]. To our knowledge, this is the second published case of DM associated with a solid pseudopapillary pancreatic tumor and the first linked to anti–TIF1γ positivity [9].

Solid pseudopapillary tumor represents < 3% of exocrine pancreatic neoplasms and ~ 5% of pancreatic cystic tumors, predominantly affecting young women [21]. Classic MRI findings include the absence of septa, a predilection for the body or tail, and heterogeneous solid–cystic components, with the solid portion iso- or hypointense on T1-weighted images and mildly hyperintense on T2-weighted images [22]. In this case, the uncinate process location is rare (< 1%) [21]. Prognosis is favorable, with 5-year survival ~ 97% and low recurrence after complete resection [23, 24].

Approximately 90% of these tumors harbor CTNNB1 exon three mutations that encode β-catenin [25]. Wnt/β-catenin pathway dysregulation is a well-known oncogenic mechanism [26]. TIF1γ regulates β-catenin ubiquitination with protein kinase Cδ [27]. TIF1γ can act as a tumor neoantigen; mutations occur in < 1% of cancers and are associated with anti–TIF1γ DM, although no direct oncogenic role of the autoantibody has been demonstrated [28]. This may partially explain tumor–autoantibody interactions in this patient.

In this case, the rapid normalization of muscle enzyme levels and the improvement of cutaneous lesions following tumor resection strongly support a paraneoplastic etiology. Regarding the persistence muscle weakness despite surgical intervention and immunomodulatory therapy, the concomitant infection with *Mycobacterium tuberculosis* served as a significant confounding factor. This infection, coupled with the sequelae of prolonged intensive care unit stay (ICU-acquired weakness) likely hindered the patient`s clinical recovery and contributed to her deterioration. Furthermore, published case reports of paraneoplastic dermatomyositis describe a heterogeneous clinical course following oncological treatment, with responses ranging from immediate postoperative remission to a partial, delayed, or even negligible improvement [29–33].

Teboul et al. analyzed a cohort of 73 patients with paraneoplastic dermatomyositis and found reported a median time to achieve complete clinical response (CRR) of 1.42 years (95% CI: 1.08–2.75) following tumor resection. Notably, only 49.3% of the patients achieved CRR during the follow-up period [34]. These findings support that the absence of immediate or early clinical improvement does not preclude a paraneoplastic etiology, especially in patients with complex intercurrent factors such as severe infections, chronic comorbidities, or prolonged stays in ICU.

## Conclusions

DM associated with anti–TIF1γ antibodies poses significant diagnostic and therapeutic challenges, particularly due to its strong association with occult malignancy. The identification of a solid pseudopapillary pancreatic tumor in a young woman with DM and anti–TIF1γ positivity represents a exceedingly rare association that broadens the spectrum of malignancies linked to this disease.

Atypical cutaneous manifestations—pseudoangioedema, ovoid palatal patch, and generalized subcutaneous edema—serve as important clinical markers of paraneoplastic forms. Their presence should raise suspicion for an underlying neoplasm, especially when accompanied by high-risk antibodies such as anti–TIF1γ. Although rare, these findings provide a valuable diagnostic window for earlier detection of malignancy-associated DM.

This case reinforces the importance of recognizing immunologic and dermatologic patterns characteristic of high-risk DM phenotypes and highlights the inclusion of solid pseudopapillary pancreatic tumors among potential associated malignancies.

## Data Availability

Data sharing is not applicable to this article as no datasets were generated or analyzed beyond those included in the manuscript.
